# Association of *COL5A1* gene polymorphisms and risk of tendon-ligament injuries among Caucasians: a meta-analysis

**DOI:** 10.1186/s40798-018-0161-0

**Published:** 2018-10-22

**Authors:** Noel Pabalan, Phuntila Tharabenjasin, Suphawadee Phababpha, Hamdi Jarjanazi

**Affiliations:** 1 Chulabhorn International College of Medicine, Thammasat University, Pathum Thani, 12120 Thailand; 2 Environmental Monitoring and Reporting Branch, Ontario Ministry of the Environment and Climate Change, 125 Resources Road, Toronto, ON Canada

**Keywords:** *COL5A1* polymorphisms, Tendon-ligament injury, Meta-analysis

## Abstract

**Background:**

Tendons and ligaments are common sites of musculoskeletal injuries especially during physical activity. The multifactorial etiology of tendon-ligament injury (TLI) includes both genetic and environmental factors. The genetic component could render influence on TLI risk to be either elevation or reduction.

**Objective:**

Inconsistency of reported associations of the collagen type V alpha 1 chain (*COL5A1*) polymorphisms, mainly rs12722 (BstUI) and rs13946 (DpnII), with TLI warrant a meta-analysis to determine more precise pooled associations.

**Methods:**

Multi-database literature search yielded eight articles (11 studies) for inclusion. Pooled odds ratios (ORs) and 95% confidence intervals were used to estimate associations. Heterogeneity of outcomes warranted examining their sources with outlier treatment.

**Results:**

All rs12722 effects indicated reduced risk (OR < 1.0). The significant outcomes (ORs 0.59–0.77, *p* = 0.0009–0.04) in the pre-outlier analysis were non-heterogeneous (*p >* 0.10). The non-significant and heterogeneous (ORs 0.63–0.98, *p* = 0.13–0.95; up to *I*^2^ = 86%) pre-outlier rs12722 and rs13946 results became significant (ORs 0.32–0.78, *p* = 10^−5^−0.01) and heterogeneity eliminated (*I*^2^ = 0%) with outlier treatment. Significant associations (ORs 0.26–0.65, *p* = 0.002–0.03) were also observed in other *COL5A1* polymorphisms (rs71746744 and rs16399). Sensitivity analysis deemed all significant outcomes to be robust.

**Conclusions:**

In summary, *COL5A1* polymorphisms reduce the risk of TLI among Caucasians. These findings are based on the evidence of significance, homogeneity, consistency, and robustness. Additional studies are warranted to draw more comprehensive conclusions.

**Electronic supplementary material:**

The online version of this article (10.1186/s40798-018-0161-0) contains supplementary material, which is available to authorized users.

## Key points

- *COL5A1* polymorphisms reduce the risk of tendon-ligament injury among Caucasians.
- Pre-outlier reduced risk effects on tendon-ligament injury were significant in rs12722 but not in rs13946.
- Outlier treatment impacted upon the rs12722 and rs13946 polymorphism outcomes, with significance strengthened and gained, respectively.
- Non-heterogeneous significant reduced risk of tendon injury observed in rs71746744 and rs16399 obviated the need for outlier treatment.

## Background

Normal tendons and ligaments differ in function and are impacted under conditions of injury [1]. Tendon-ligament injury (TLI) includes Achilles tendon pathology (ATP), Achilles tendinopathy (AT), tennis elbow (TE), and anterior cruciate ligament rupture (ACLR). These common sites of musculoskeletal injuries are occupational and sports-related [2, 3]. ATP is a broad term that refers to AT which results from acute or repetitive mechanical loading during occupational and sporting activities [4]. TE (lateral epicondylitis) is a painful musculotendinous condition originating from the lateral epicondyle of the humerus related to overuse [5] and involves highly repetitive movements [6]. ACLR involves sprain or tear of the anterior cruciate ligament which scaffolds the bones within the knee helping to keep it stable. As over 70% of ACLR injuries are noncontact [7], it puts high risk (up to 10 times) on athletes performing sudden decelerations or changes in direction [8]. ACL ruptures are considered one of the most severe injuries sustained in sports [9]. Etiology of TLI involves genes and protein structure changes [3]. Changes in collagen composition and expression of the genes that encode for these proteins have been shown to be altered in TLI [10]. Collagen is the main component of tendons and ligaments [11]; its fibrils comprising collagen type I, III, V, VI, XI, and XIV [12]. Type V collagen may be a structurally minor player in the collagen hierarchy but is functionally prominent where it plays an important role in regulating fiber diameter as well as assembly (fibrillogenesis) of collagen fibers [13]. Type V collagen protein is encoded by the collagen type V alpha 1 chain (*COL5A1*) gene, located on the long (q) arm of chromosome 9 [14] and is expressed in both tendons and ligaments [1]. Polymorphisms in the *COL5A1* gene have been found to impact upon TLI, the most prominent being rs12722 (BstUI) and rs13946 (DpnII) which are found within the 3′-untranslated region (UTR) [15]. Other *COL5A1* polymorphisms (rs71746744, rs16399, and rs3196378) have been considered as risk modifiers [16]. Primary studies have been conducted to investigate genetic risk factors involving *COL5A1* polymorphisms in TLI, but results have been inconsistent. Inconsistency of results may be attributed to lack of statistical power because of small sample sizes. Meta-analysis synthesizes primary study data yielding an aggregate sample size to indicate raised statistical power. Therefore, we perform a meta-analysis of all available data on *COL5A1* polymorphisms and their relationship with TLI so that we could obtain better estimates of associations.

## Methods

### Selection of studies

We searched MEDLINE using PubMed, Science Direct, and Google Scholar for association studies as of April 15, 2018. The terms used were “type V collagen,” “*COL5A1*,” “ligament injury,” “tendon injury,” and “polymorphism” as medical subject heading and text, restricted to English. References cited in the retrieved articles were also screened manually to identify additional eligible studies. Inclusion criteria were (i) case–control studies evaluating the association between *COL5A1* polymorphisms and risk for TLI, (ii) sufficient genotype/allele frequency data presented to calculate the odds ratios (ORs) and 95% confidence intervals (CIs), and (iii) participants were either athletes or non-athletes. Exclusion criteria were (i) non-English articles and (ii) studies whose genotype or allele frequencies were unavailable or, when they are, combined with other polymorphisms, preventing proper data extraction.

### Data extraction

Two investigators (NP and PT) independently extracted data and arrived at a consensus. Extracted data were tabulated; when needed, we contacted authors of the original articles to request for additional information. The following information were obtained from each publication: first author’s name, published year, country of origin, ethnicity, TLI and *COL5A1* polymorphism type, and basis for matching (Table 1). Departures of genotypic frequencies from the Hardy–Weinberg Equilibrium (HWE) in control subjects were determined with the *χ*^2^ test.

**Table 1:** Characteristics of the included studies that examined the association of *COL5A1* polymorphisms with TLI studies

K	First author	[R]	*n*	Year	Country	TLI	*COL5A1* Polymorphisms	Tissue Sources	UM	BM	CB Score
1	Abraham	[25]	2	2013	SA/AU	AT	rs71746744/rs16399	Blood	Yes	Ag/He	8
2	Altinisik	[26]	1	2015	TUR	TE	rs12722, rs13946	Blood	Yes	Ag/Sx	7
3	Brown	[27]	1	2016	UK	ATP	rs12722/rs3196378/rs71746744	Oral	Yes	Ag/G	8
4	Mokone	[28]	1	2006	SA	ATP	rs12722	Blood	Yes	Ag/Sx	8
5	O’Connell	[29]	2	2014	SA/POL	ACLR	rs12722	Blood	Yes	Ag/Sx/We	9
6	Posthumus	[30]	1	2009	SA	ACLR	rs12722/rs13946	Blood	Yes	Ag	6
7	September	[31]	2	2009	SA/AU	AT	rs12722/rs13946/rs3196378	Blood	Yes	Ag/G	8
8	Stepien–Slodkowska	[32]	1	2015	POL	ACLR	rs12722/rs13946	Oral	Yes	NM	9

*K* number designation of the article, *[R]* reference, *n* number of studies per article, *SA* South Africa, *AU* Australia, *TUR* Turkey, *UK* United Kingdom, *POL* Poland, *TLI* tendon-ligament injury, *AT* achilles tendinopathy, *TE* tennis elbow, *ATP* achilles tendon pathology, *ACLR* anterior cruciate ligament rupture, *rs12722* BstUI, *rs13946* DpnII, *UM* used matching, *BM* basis for matching, *Ag* age, *He* height, *Sx* sex, *G* geography, *We* weight, *NM* no mention, *CB* Clark–Baudouin

### Modifier treatment and subgrouping

Additional file 1: Table S1 shows the sample sizes, number of cases and controls, and genotype frequencies, including the minor allele (maf) and *p* values for HWE. Confining the analyses to HWE-compliant studies constituted modifier treatment. Subgrouping was based on injury type (tendon or ligament).

### Quality assessment of the studies

We used the Clark–Baudouin (CB) scale to evaluate methodological quality of the included studies [17]. We found this scale most appropriate because it uses criteria such as *p* values, power, corrections for multiplicity, comparative sample sizes between cases and controls, use of the HWE, and genotyping methods. CB scores range from 0 (worst) to 10 (best) where scoring is based on quality (low < 5, moderate 5–7, and high ≥ 8).

### Meta-analysis

Examining five *COL5A1* polymorphisms (rs12722, rs13946, rs71746744, rs16399, and rs3196378) warranted the use of a common notation indicating variant (*var*) and wild-type (*wt*) alleles. Additional file 1: Table S1, however, details the genotypes for each of the five *COL5A1* polymorphisms. After estimating TLI risk (OR) for each study, pooled ORs with 95% CIs were calculated for the following genetic models: (i) homozygous: (*var*–*var* and *wt*–*wt*) genotypes compared with *wt*–*wt*, (ii) recessive: (*var*–*var* versus *wt*–*var*+*wt*–*wt*), (iii) dominant: (*wt–wt* versus *wt–var+var–var*), and (iv) codominant: (*var* versus *wt*). To compare effects on the same baseline, we used raw data for genotype frequencies to calculate pooled ORs, using either fixed [18] or random [19] effects models. Heterogeneity between studies was (i) estimated with the *χ*^2^-based Q test [20], (ii) quantified with the *I*^2^ statistic which measures degree of inconsistency among studies [21], and (iii) its sources (outliers) detected with the Galbraith plot [22], then subjected to outlier treatment which involves elimination of the outliers followed by reanalysis. Sensitivity analysis, which involves omitting one study at a time and recalculating the pooled ORs, was used to test for robustness of the summary effects. Robustness indicates that the pooled effects are stable, unaltered even when each study is removed. Publication bias was not examined because the qualitative and quantitative tests have low sensitivity when the number of studies is < 10 [23]. Data were analyzed using Review Manager 5.3 (Cochrane Collaboration, Oxford, England), SIGMASTAT 2.03 and SIGMAPLOT 11.0 (Systat Software, San Jose, CA, USA). Two-sided *p* values of < 0.05 were considered significant except in estimations of heterogeneity. Given the low power of the *χ*^2^-based Q test for heterogeneity, the *p* value was set at < 0.10 [24].

## Results

### Search outcomes

**Fig. 1:**
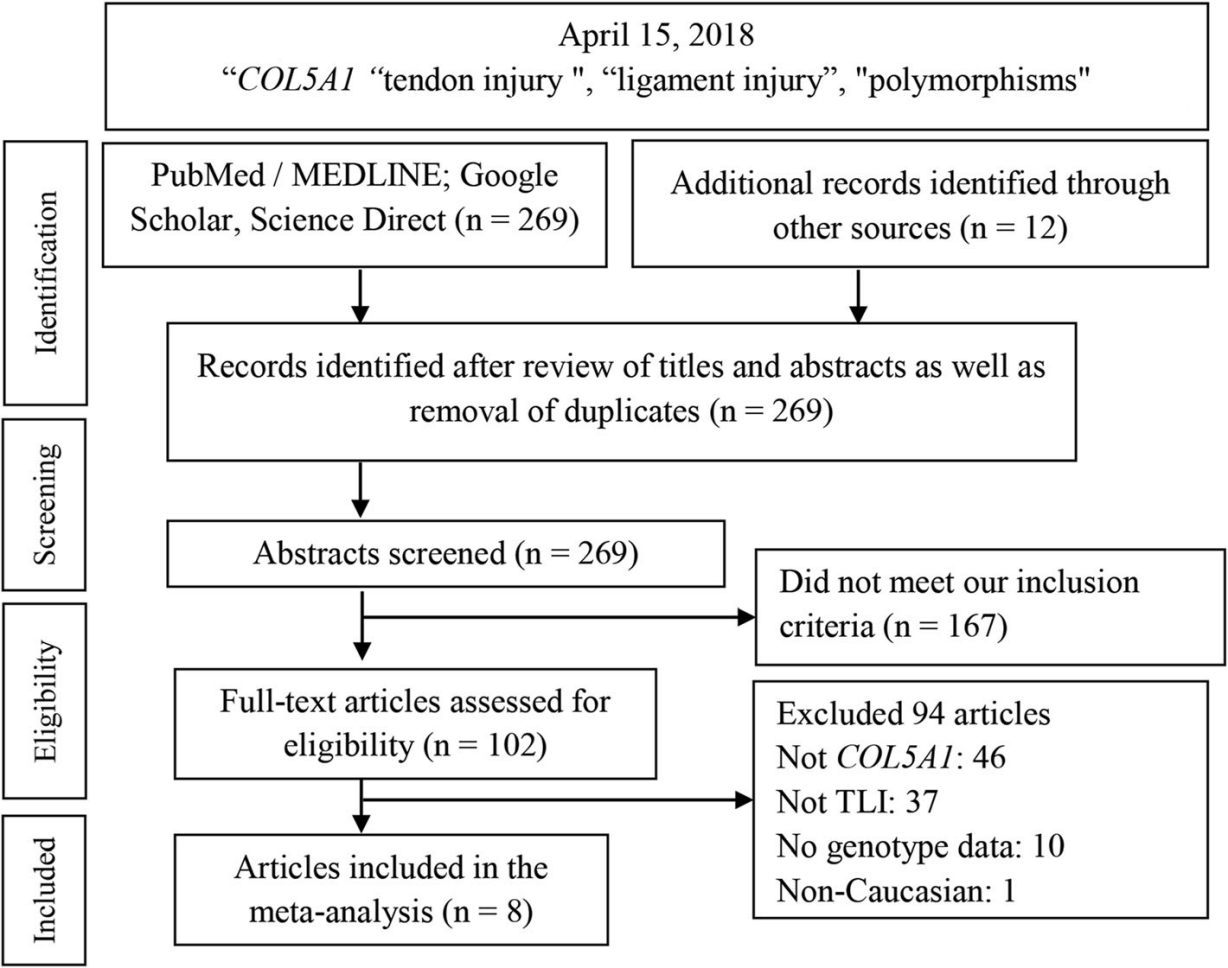
Summary flowchart of literature search. TLI tendon-ligament injury

Figure 1 outlines the study selection process in a flowchart following PRISMA (Preferred Reporting Items for Systematic Reviews and Meta-Analyses) guidelines. A total of 281 citations during the initial search was subjected to a series of omissions that eventually yielded eight articles for inclusion [25–32].

### Study characteristics

Table 1 shows the year range of studies from 2006 to 2016. We confined our meta-analysis to Caucasians only in order to reduce the likelihood of confounding by population stratification. Injury type subgroups were tendon (AT/ATP/TE) and ligament (ACLR). CB scores for the mean (7.88 ± 0.99), median (8.0), and range (6–9) indicate that methodological quality of the component studies was high. Additional file 1: Table S1 shows the quantitative features of the component studies under each of the five polymorphisms. Independent data from three articles [25, 29, 31] (on account of geography), put the number of studies to nine, five, three, two, and three for rs12722, rs13946, rs71746744, rs16399, and rs3196378, respectively. Respective aggregate sample sizes (case/control) for these polymorphisms are 1234/1667, 546/934, 191/299, 120/254, and 270/520. Assuming small effect size (*d* = 0.20) and an *α* level of 0.05 (two-tail), statistical powers are adequate for rs12722 (99%) and rs13946 (96%). Two studies [26, 28] reported data on rs12722 *A1* and *A2* alleles which are BstUI products of restriction fragment length polymorphism due to two single-nucleotide polymorphism substitutions, the *A2* allele referring to the variant C allele [33]. Extracted separately, these data yielded highly significant *p* values for HWE (up to 10^−5^). We minimized this by combining the *A1* and *A2* data. Three studies from two articles were HWE-non-compliant [26, 31]. The PRISMA checklist provides detailed description of this meta-analysis (Additional file 2: Table S2).

### rs12722 and rs13946 effects

Pooled effects (*p* < 0.05) indicating reduced (protective) risk (OR < 1.0) were observed in all significant ORs and non-significant ORs that were altered with outlier treatment as well as all post-outlier outcomes (Table 2). All post-outlier outcomes were also non-heterogeneous (fixed-effects). Outlier treatment induced the following outcomes: (i) significance either acquired or elevated, (ii) heterogeneity either reduced or eliminated, and (iii) narrowing of CIs indicating increased precision.

**Table 2:** Summary effects in the overall, modifier (HWE), and subgroup (tendon/ligament) analyses of *COL5A1* with TLI

Comparison [R] genetic model	*n*	Test of association			Test of heterogeneity		
		OR	95% CI	*P* ^a^	*P* ^b^	*I*^2^ (%)	AM
rs12722 All [26–32]							
H	9	*0.69*	*0.54–0.89*	*0.003*	0.25	21	FE
R	9	*0.72*	*0.52–0.99*	*0.04*	0.05	48	RE
D	9	0.90	0.68–1.21	0.49	0.001	69	RE
C	9	0.87	0.69–1.10	0.24	0.0001	75	RE
rs13946 All [26, 30–32]							
H	5	0.83	0.33–2.08	0.69	0.006	72	RE
R	5	0.79	0.35–1.78	0.57	0.02	67	RE
D	5	1.12	0.78–1.63	0.54	0.02	65	RE
C	5	1.01	0.76–1.34	0.94	0.03	63	RE
rs12722 HWE [27–32]							
H	7	*0.68*	*0.52–0.88*	*0.004*	0.13	39	FE
R	7	0.76	0.53–1.09	0.13	0.04	55	RE
D	7	0.86	0.66–1.12	0.27	0.05	52	RE
C	7	0.88	0.66–1.17	0.37	10^−5^	79	RE
rs13946 HWE [26, 30–32]							
H	4	1.03	0.39–2.72	0.95	0.02	70	RE
R	4	1.00	0.44–2.27	1.00	0.05	61	RE
D	4	1.15	0.72–1.83	0.56	0.01	73	RE
C	4	1.06	0.76–1.46	0.74	0.03	68	RE
rs12722 Tendon [26–28, 31]							
H	5	0.87	0.59–1.27	0.46	0.11	47	FE
R	5	0.81	0.42–1.57	0.53	0.009	70	RE
D	5	0.84	0.55–1.29	0.43	0.01	73	RE
C	5	0.98	0.62–1.55	0.95	10^−5^	86	RE
rs13946 Tendon [26, 31]							
H	3	0.70	0.14–3.56	0.66	0.001	85	RE
R	3	0.63	0.16–2.58	0.52	0.005	81	RE
D	3	1.15	0.63–2.12	0.65	0.01	78	RE
C	3	0.97	0.58–1.61	0.90	0.006	81	RE
rs12722 Ligament [29, 30, 32]							
H	4	*0.59*	*0.43–0.82*	*0.001*	0.96	0	FE
R	4	*0.65*	*0.49–0.86*	*0.003*	0.80	0	FE
D	4	0.99	0.66–1.50	0.96	0.03	68	RE
C	4	*0.77*	*0.66–0.90*	*0.0009*	0.95	0	FE
rs13946 Ligament [30, 32]							
H	2	1.07	0.51–2.26	0.85	0.39	0	FE
R	2	1.10	0.54–2.24	0.80	0.22	34	FE
D	2	1.04	0.74–1.46	0.82	0.16	48	FE
C	2	1.04	0.80–1.35	0.79	0.53	0	FE

Cut-offs for *P*^*a*^ and *P*^*b*^ are < 0.05 and 0.10, respectively. Values in italics indicate significant associations

*H* homozygous, *R* recessive, *D* dominant, *C* codominant, *n* number of studies, *[R]* references, *OR* odds ratio, *CI* confidence interval, *P*^a^ *p* value for association, *P*^b^ *p* value for heterogeneity, *AM* analysis model, *HWE* Hardy–Weinberg Equilibrium, *FE* fixed-effects, *RE* random-effects

#### Overall

Our hypothesis-driven approach indicates associations of *COL5A1* with TLI. Table 2 shows significant pre-outlier effects in rs12722 but not rs13946. These associations were observed in the overall (ORs 0.69–0.72, *p* = 0.003–0.04) and modifier (OR 0.68, *p* = 0.004) analyses. Non-significant heterogeneous pooled effects in rs12722 and rs13946 (overall and HWE: ORs 0.76–0.90, *p* = 0.13–0.69) were altered to significance with outlier treatment, outcomes of which are summarized in Table 3. These outcomes show gain in significance seen in the overall analysis of rs12722 (ORs 0.72–0.78, *p* = 0.0001–0.0003) and rs13946 (ORs 0.35–0.37, *p* = 0.009–0.01), modifier (HWE) outcomes in rs12722 (ORs 0.67–0.76, *p* = 10^−5^−0.004).

**Table 3:** Post-outlier analysis outcomes of the *COL5A1* polymorphism associations with TLI

Comparison genetic model	*n*	[R]	Test of association			Test of heterogeneity			Outlier treatment effect
			OR	95% CI	*P* ^a^	*P* ^*b*^	*I*^2^ (%)	AM	
rs12722 all									
R	8	[26, 27, 29, 31, 32]	*0.65*	*0.52–0.81*	*0.0002*	0.19	30	FE	RH, ES
D	7	[26–29, 31, 32]	*0.72*	*0.61–0.86*	*0.0003*	0.88	0	FE	EH, GS
C	8	[26, 28–32]	*0.78*	*0.69–0.87*	*0.0001*	0.63	0	FE	EH, GS
rs13946 all									
H	3	[30, 31]	*0.37*	*0.17–0.82*	*0.01*	0.73	0	FE	EH, GS
R	3	[30, 31]	*0.35*	*0.16–0.77*	*0.009*	0.86	0	FE	EH, GS
D	4	[30–32]	0.95	0.74–1.22	0.69	0.36	8	FE	RH
C	4	[30–32]	0.90	0.74–1.10	0.30	0.36	6	FE	RH
rs12722 HWE									
R	6	[27, 29–32]	*0.67*	*0.53–0.86*	*0.002*	0.14	40	FE	RH. GS
D	6	[27–32]	*0.76*	*0.63–0.92*	*0.004*	0.96	0	FE	EH, GS
C	6	[28–32]	*0.76*	*0.66–0.86*	*10* ^*−5*^	0.94	0	FE	EH, GS
rs13946 HWE									
H	3	[31]	0.73	0.38–1.38	0.33	0.16	46	FE	RH
R	3	[31]	0.77	0.41–1.43	0.40	0.11	55	FE	RH
D	3	[31]	0.93	0.71–1.24	0.64	0.20	37	FE	RH
C	3	[31]	0.92	0.74–1.16	0.49	0.23	32	FE	RH
rs12722 tendon									
R	3	[26, 31]	*0.45*	*0.28–0.72*	*0.0008*	0.45	0	FE	EH, GS
D	4	[26–28, 31]	*0.66*	*0.52–0.84*	*0.0006*	0.76	0	FE	EH, GS
C	4	[26–28, 31]	*0.78*	*0.65–0.94*	*0.008*	0.18	38	FE	RH, GS
rs13946 tendon									
H	2	[31]	*0.32*	*0.13–0.79*	*0.01*	0.69	0	FE	EH, GS
R	2	[31]	*0.32*	*0.13–0.78*	*0.01*	0.79	0	FE	EH, GS
D	2	[31]	0.85	0.59–1.24	0.40	0.39	0	FE	EH
C	2	[31]	0.75	0.55–1.02	0.06	0.58	0	FE	EH
rs12722 ligament									
D	3	[29, 30, 32]	0.8	0.62–1.04	0.10	0.97	0	FE	EH

Cut-offs for *P*^a^ and *P*^b^ are < 0.05 and 0.10, respectively. Values in italics indicate significant associations

*H* homozygous, *R* recessive, *D* dominant, *C* codominant, *n* number of studies, *[R]* References, *OR* odds ratio, *CI* confidence interval, *P*^*a*^ *p*-value for association, *P*^b^ *p* value for heterogeneity, *AM* analysis model, *HWE* Hardy–Weinberg Equilibrium, *FE* fixed-effects, *RH* reduced heterogeneity, *ES* elevated significance, *EH* eliminated heterogeneity, *GS* gained significance

#### *Mechanism of outlier treatment in* rs12722

**Fig. 2:**
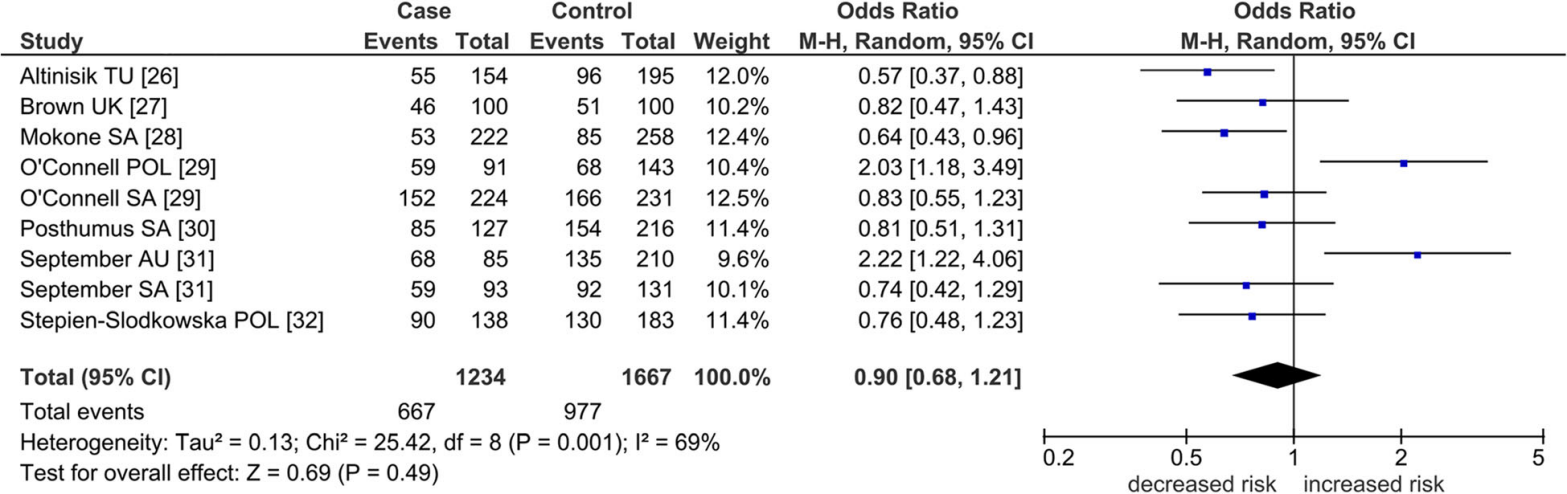
Summary association of *COL5A1* rs12722 polymorphism with TLI studies in the dominant model. Numbers in brackets under the study column indicate references. Squares indicate the odds ratio (OR) in each study, with square sizes directly proportional to the weight contribution (%) of the study. Horizontal lines on each side of the squares represent 95% confidence intervals (CIs). The random-effects model was used on account of the presence of heterogeneity (*p* = 0.001, *I*^2^ = 69%). M–H Mantel–Haenszel; df degree of freedom; TU Turkey; UK United Kingdom; POL Poland; SA South Africa; AU Australia

**Fig. 3:**
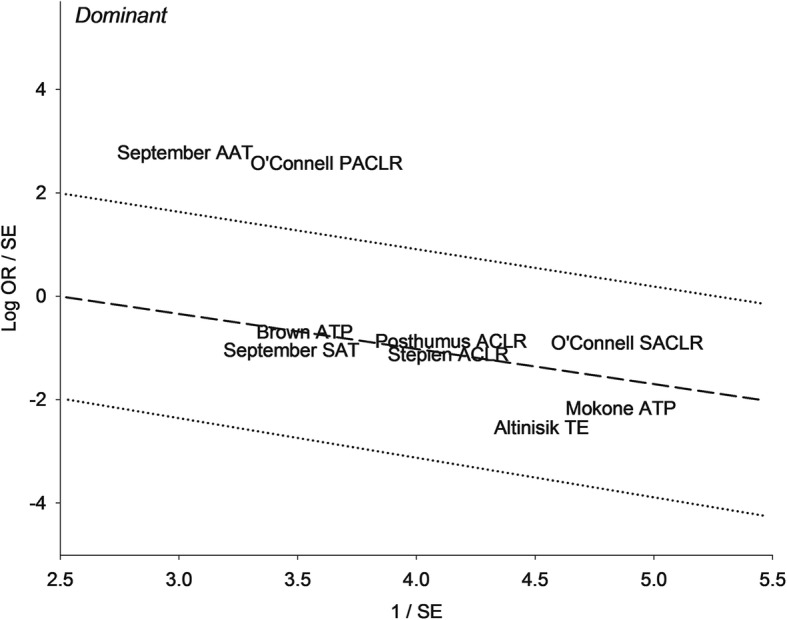
Galbraith plot analysis of *COL5A1* rs12722 polymorphism in the dominant model. The two studies found above the + 2 confidence limit are identified as outliers. OR, odds ratio; SE, standard error; AAT, Australia Achilles Tendinopathy; PACLR, Poland Anterior Cruciate Ligament Rupture; SAT, South Africa Achilles Tendinopathy; SACLR, South Africa Anterior Cruciate Ligament Rupture; ATP, Achilles Tendinopathy; TE, Tennis elbow

**Fig. 4:**
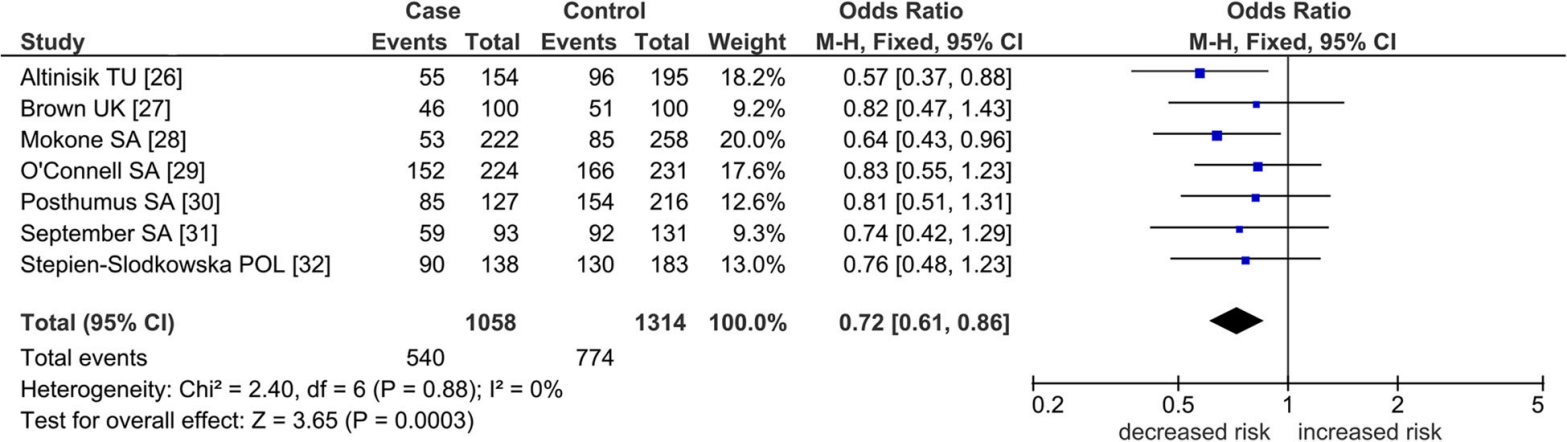
Post-outlier association of *COL5A1* rs12722 polymorphism with TLI studies in the dominant model. Numbers in brackets under the study column indicate references. Squares indicate the odds ratio (OR) in each study, with square sizes directly proportional to the weight contribution (%) of the study. Horizontal lines on each side of the squares represent 95% confidence intervals (CIs). The fixed-effects model was used on account of zero heterogeneity (*p* = 0.88, *I*^2^ = 0%). M–H Mantel–Haenszel; df degree of freedom; TU, Turkey; UK, United Kingdom; SA, South Africa; POL, Poland

Operation of outlier treatment is visualized in Figs. 2, 3, and 4 for rs12722 in the dominant model. In Fig. 2, the pooled effect is non-significant (OR 0.90, *p* = 0.49) and heterogeneous (*I*^2^ = 69%). Sources of this heterogeneity were identified with the Galbraith plot visualized in Fig. 3 which shows the two outlying studies [29, 31] located above the + 2 confidence limit. Figure 4 shows the post-outlier value of acquired significance (OR 0.72, *p* = 0.0003) and eliminated heterogeneity (*I*^2^ = 0%).

#### Tendon/ligament subgroups

Table 2 shows that rs12722 ligament pooled ORs were the only significant subgroup outcomes in the pre-outlier analyses (ORs 0.59–0.77, *p* = 0.0009–0.003). Non-significant tendon subgroup outcomes (rs12722: ORs 0.81–0.98, *p* = 0.43–0.95; rs13946: ORs 0.63–0.70, *p* = 0.52–0.66) were altered to significance (rs12722: ORs 0.45–0.78, *p* = 0.0006–0.008; rs13946: ORs 0.32, *p* = 0.01) following outlier treatment (Table 3).

### Effects of the other *COL5A1* polymorphisms

Table 4 shows associations of rs71746744 (ORs 0.45–0.65, *p* = 0.003–0.03) and rs16399 (ORs 0.26–0.58, *p* = 0.002–0.02) with tendon injuries only but not rs319378 (ORs 0.70–1.22, *p* = 0.06–0.77). These associations were either non-heterogeneous (*I*^2^ = 12–46%) or homogeneous (*I*^2^ = 0%).

**Table 4:** Summary outcomes of associations in rs71746744, rs16399, and rs3196378 COL5A1 polymorphisms with tendon injury

Comparison [R] genetic model	Test of association				Test of heterogeneity		
	*n*	OR	95% CI	*P* ^a^	*P* ^b^	*I*^2^ (%)	AM
rs71746744 [25, 27]							
H	3	*0.45*	*0.22–0.95*	*0.03*	0.31	13	FE
R	3	0.57	0.28–1.17	0.13	0.32	12	FE
D	3	*0.57*	*0.39–0.83*	*0.004*	0.70	0	FE
C	3	*0.65*	*0.48–0.87*	*0.003*	0.41	0	FE
rs16399 [25]							
H	2	*0.26*	*0.10–0.69*	*0.007*	0.38	0	FE
R	2	*0.31*	*0.12–0.81*	*0.02*	0.40	0	FE
D	2	*0.57*	*0.37–0.88*	*0.01*	0.42	0	FE
C	2	*0.58*	*0.41–0.82*	*0.002*	0.23	30	FE
rs319378 [27, 31]							
H	3	0.93	0.58–1.49	0.77	0.76	0	FE
R	3	0.70	0.49–1.01	0.06	0.50	0	FE
D	3	1.22	0.84–1.77	0.29	0.16	46	FE
C	3	0.83	0.67–1.02	0.08	0.22	34	FE

Cut-offs for *P*^a^ and *P*^b^ are < 0.05 and 0.10, respectively. Values in italics indicate significant associations

*H* homozygous, *R* recessive, *D* dominant, *C* codominant, *n* number of studies, *[R]* References, *OR* odds ratio, *CI* confidence interval, *P*^a^ *p* value for association, *P*^b^ *p* value for heterogeneity, *AM* analysis model, *FE* fixed-effects

### Heterogeneity analysis

Pre-outlier ORs were generally heterogeneous (*p*_heterogeneity_ < 0.10). Outlier treatment impacts upon heterogeneity by reducing (*p*_heterogeneity_ > 0.10) or eliminating it (*I*^2^ = 0%). Table 2 shows six significant pooled ORs, five of which are non-heterogeneous (fixed-effects). Of the five, three (60%) have zero heterogeneity (*I*^2^ = 0%). Table 3 shows 13 significant pooled ORs, all of which are non-heterogeneous (fixed-effects). Of the 13, 10 (77%) have zero heterogeneity (*I*^*2*^ = 0%).

### Sensitivity analysis

This treatment was applied in all comparisons and stratified by genetic model with emphasis on significant effects (*).Table 5 summarizes our sensitivity analysis findings. The total number of significant effects across comparisons and genetic models is indicated by (S). Comparisons that were robust are labeled as such (robust) and those that were not are identified by reference number. Reference numbers reduce robustness of the comparisons. The total number of robust comparisons is indicated by (B), and the total number of identified reference numbers is indicated by (A). This approach to sensitivity treatment allows identification of the most and least robust comparisons and genetic models. Aggregate robustness was based on the least number of A and most counts for B and S. Thus, the most robust comparisons were rs12722 (pre- and post-outlier outcomes) and rs71746744 effects and in terms of genetic model, recessive and codominant effects.

**Table 5:** Sensitivity analysis for all *COL5A1* comparisons

Comparison	Genetic model				S	A	B
	Homozygous	Recessive	Dominant	Codominant			
All							
rs12722	Robust*	Robust*	Robust	Robust	2	0	4
rs13946	[31]^a^ [31]^b^	[31]^b^	[26]	[26, 30]	0	4	0
Modifier							
rs12722 HWE	Robust*	Robust	Robust	Robust	1	0	4
rs13946 HWE	[26, 32]	[26, 32]	[26]	[31]^a^	0	3	0
Subgroup							
rs12722 tendon	[31]^b^	[31]^b^	Robust	[26, 28] [31]^b^	0	3	1
rs13946 tendon	[31]^a^	[26]	Robust	[31]^a^ [31]^b^	0	3	1
rs12722 ligament	Robust*	Robust*	[29, 30, 32]	Robust*	3	3	3
rs13946 ligament	Robust	Robust	[30]	[30]	0	1	2
Other *COL5A1* polymorphisms							
rs71746744	Robust*	Robust	Robust*	Robust*	3	0	4
rs16399	Robust*	Robust*	Robust*	Robust*	4	0	4
rs3196378	[27]	Robust	Robust	Robust	0	1	3
Post-outlier							
All							
rs12722	–	Robust*	Robust*	Robust*	3	0	3
rs13946	Robust*	Robust*	[31]^a^	Robust	2	1	3
Modifier							
rs12722 HWE	–	Robust*	Robust*	Robust*	3	0	3
rs13946 HWE	[31]^a^	[31]^a^	[31]^a^	[31]^a^	0	1	0
Subgroup							
rs12722 tendon	[31]^b^	Robust*	Robust*	Robust*	3	1	3
rs13946 tendon	Robust*	Robust*	[31]^a^	Robust	2	1	3
rs12722 ligament	–	–	[29, 30, 32]	–	0	3	0
rs13946 ligament	[30]	[32]	[30]	[30]	0	2	0
S	7	8	5	6			
A	6	4	5	5			
B	8	12	10	11			
Total number of comparisons	16	18	19	18			

*HWE* Hardy–Weinberg Equilibrium, *S* number of significant associations, *A* number of non-redundant references that contributed to instability, *B* number of robust findings

*Significant associations

[31]^a^ AU; [31]^b^ SA

## Discussion

### Summary of findings

Applying meta-analytical techniques is methodologically complex. This complexity arises from interpreting outcomes resulting from the combined applications of genetic modeling, modifier, outlier, and subgroup analyses as well sensitivity treatment. These considered, rs12722 was more significant (*p* < 0.05) than rs13946. For both polymorphisms, the most associated models with TLI were homozygous and recessive more than dominant/codominant. On the whole, this meta-analysis delineated which polymorphisms in the *COL5A1* gene have associations with TLI (rs12722, rs71746744, rs16399) and those that do not (rs319378) as well as those that were altered (rs12722 and rs13946) with meta-analytical treatment (outlier analysis) such that significance was either intensified or gained. The main finding of this study points to significant reduced risk effects (all ORs < 1.0), up to 41% and 55%, pre- and post-outlier, respectively, in rs12722 and up to 68% in rs13946, post-outlier only. Outlier treatment impacted upon heterogeneity, significance, and precision of outcomes, seen in the overall, modifier, and subgroup analyses. Significant effects (up to 69%) of the other *COL5A1* polymorphisms were consistent and homogeneous (most had *I*^2^ = 0%) indicating associations of rs71746744 and rs16399 with tendon injury. The combination of significance, consistency, homogeneity, and increased precision of pooled effects in these comparisons improved our findings.

### Comparisons with other meta-analysis

We compare our findings with a recent (March 2018) meta-analysis [34] which examined rs12722 only, compared to five *COL5A1* polymorphisms in ours. This additional array enabled us to contrast and compare effects of rs12722 with rs13946, not only in the overall analysis but also in the modifier and subgroup outcomes. Caucasian rs12722 comparisons between Lv et al. [34] and ours were based on sample sizes of 1381 and 2677, respectively. Recessive outcomes from these two meta-analyses had contrasting effects. This led us to examine qualitative and quantitative differences at the data level of the primary studies in rs12722 among Caucasians. First, both meta-analyses had six studies in common [26–30, 32]. The previous study [34] included Raleigh et al. [35] who examined interaction between matrix metalloproteinase 3 (*MMP3*) and *COL5A1* but did not differentiate genotype data between these two genes, which was our reason for excluding it in our meta-analysis. Of note, *MMP3* was genotyped but not *COL5A1*. Second, we had two added studies [25, 31] which were not in the previous study [34]. Third, the recessive model was defined differently, *TT* versus *TC*+*CC* for the previous study [34] and *var*–*var* versus *wt*–*var*+*wt*–*wt* in our study. Given this contrast, calculations of the ORs would inevitably have taken diverging outcomes. Thus, OR risks were increased for the previous study [34] and reduced for ours. Other major differences were as follows: (i) our use of outlier treatment but not the previous study [34]; (ii) they tested publication bias, we did not; and (iii) our use of standard genetic models versus model-free approach for Lv et al. [34].

### Comparisons with primary studies

Comparing our reduced risk pooled findings with the component study-specific ORs shows the following for the rs12722 *CC* genotype: (i) decreased AT risk of up to 62% in different populations (Australia and South Africa) [31], (ii) reduced ACLR risk from three studies [29, 30, 32], and (iii) contrasting effects of the *A2* and *A1* alleles in rs12722 which elicited protective and increased risks in two studies, respectively [26, 28]. In rs12722, individuals with the *CC* genotype had a significantly decreased risk of developing AT compared with those with a *T* allele in either *TT* or *TC* genotypes [31].

Genetic variations in the *COL5A1* gene 3′-UTR region affect mRNA stability and its export from the nucleus after transcription where regulatory sequences control gene expression at the posttranscriptional level [25]. Therefore, mutations or single-nucleotide variations within this region may alter mRNA secondary structure and thus protein characteristics [36]. Functionally, the rs12722 and rs71746744 variants are believed to alter stability of the *COL5A1* mRNA [25]. Abrahams et al. [25] investigated other variants in the 3′-UTR of *COL5A1* gene where rs71746744 and rs16399 were found to have significant association with AT. The rs71746744 variant *del/del* genotype was found to be associated with reduced risk of AT. Furthermore, they surmised linkage of rs12722 with rs71746744 and rs16399 [25] which suggest that the protective effects observed in this meta-analysis might be attributed to any or all of these three polymorphisms.

Reported findings on the role of genetic variants in TLI have differed between studies. Several methodological problems may explain the discrepancies, including limited statistical power, unrecognized confounding factors, misleading definition of phenotypes, and stratification of populations [17]. Reporting study-specific effects of *COL5A1* polymorphisms ranged from presence to absence of associations. In their presence, risk effects for the variant genotype were increased or reduced, significant or not. Meta-analysis, however, gives more information in reporting effects for *COL5A*. These involve exploring magnitude and precision of effects, as well as consistency and stability, all in consideration of heterogeneous outcomes. These features raise the levels of evidence to support conclusions on the associations of *COL5A1* polymorphisms with TLI.

### Strengths and limitations

Interpreting our findings should be contextualized in view of its strengths and limitations. Limitations of our study include the following: (i) we did not examine gender effects due to insufficiency of data. Nevertheless, Posthumus et al. [30] examined gender differences of the *CC* genotype among ACLR participants; (ii) *A1* and *A2* alleles for BstUI were not examined separately, but instead, we combined them which may have sacrificed precision of outcomes, given the reported contrasting effects of these two alleles in the literature [26, 28]; (iii) linkage disequilibrium was reported for the *COL5A1* polymorphisms in the component studies [25, 31, 32], which may have introduced bias [37] by masking identity of the true causal variant; and (iv) the low number of studies (*n* = 2–3) and underpowered status (58% and 44%) warrant caution in interpreting the significant effects of rs71746744 and rs16399.

On the other hand, these are the following strengths of the meta-analysis: (i) confining our meta-analysis to Caucasians rendered epidemiological homogeneity to the study which thus excludes potential confounding effects of population stratification; (ii) non-HWE-compliant studies were a minority which minimizes the issue of genotyping errors thus avoiding methodological weaknesses in the summary outputs [38]. Besides, confining our analyses to HWE-compliant studies did not materially alter the outcomes in all genetic models; (iii) overall methodological quality (determined by CB) of the included studies was high; (iv) aggregate case/control totals for rs12722 and rs13946 show that the significant findings from these two polymorphisms have statistical powers of 99% and 96%, respectively; (v) all controls were defined as healthy; (vi) most (75%) tissue sources were blood; (vii) all controls were matched with cases, with 88% based on age; and (viii) sensitivity treatment deemed the significant outcomes to be robust.

## Conclusions

The importance of our results is underpinned by the fact that each component study in this meta-analysis lacked adequate statistical power, but when combined using meta-analysis, clear reduced risk associations of *COL5A1* polymorphisms with TLI are uncovered. Genetic structure of the homozygous and recessive models point to the variant allele as protectively associated with TLI. TLI is a complex condition involving interactions of several genetic and non-genetic risk factors. Gene-gene and gene-environment interactions have been reported to have roles in the associations of *COL5A1* polymorphisms with TLI [3]. None of the eight included articles mentioned gene-environment interaction, but haplotype analysis has been addressed in the component studies [25, 27, 29, 31, 32]. However, it should be emphasized that phenotypic variations between tendon (AT, ATP, and TE) and ligament (ACLR) engender different aetiologies. Additional well-designed studies that explore other ethnic groups based on sample sizes commensurate with detection of small genotypic risks would allow more definitive conclusions about the association of *COL5A1* polymorphisms and TLI. Injury-related issues in sports medicine beg comprehensive investigation of its risks. Our contribution is the synthesis approach offered by meta-analysis in elevating the level of evidence. Given the focus of this study, we hope to have clarified the genetic risks posed by *COL5A1* polymorphisms to TLI. The mainly protective findings of *COL5A1* rs12722 and rs13946 polymorphisms may be modest given our focus on just one gene. However, the evidence we present hopes to contribute to better understanding of the genetic nature of TLI.

## Additional files

Additional file 1: **Table S1.** Quantitative characteristics of the included studies that examined the association of *COL5A1* polymorphisms with TLI studies. (DOCX 57 kb)

Additional file 2: **Table S2.** PRISMA checklist. (DOCX 30 kb)

## Abbreviations

*: Significant associations
[R]: References
3′ UTR: 3′ Untranslated region
A: Number of non-redundant references that contributed to instability
AAT: Australia Achilles Tendinopathy
ACLR: Anterior Cruciate Ligament Rupture
Ag: Age
AM: Analysis model
AT: Achilles tendinopathy
ATP: Achilles tendon pathology
AU: Australia
B: Number of robust findings
BM: Basis for matching
C: Codominant
CB: Clark–Baudouin
CI: Confidence interval
*COL5A1*: Collagen type V alpha 1 chain
D: Dominant
*d*: Measure of the effect side
EH: Eliminated heterogeneity
ES: Elevated significance
FE: Fixed-effects
G: Geography
GS: Gained significance
H: Homozygous
He: Height
HWE: Hardy–Weinberg Equilibrium
*I*^2^: Measure of variability between studies
K: Number designation of the article
maf: Minor allele frequency
*MMP3*: Matrix metalloproteinase 3
*n*: Number of studies
NM: No mention
OR: Odds ratio
*p*: *p* value
*P*^a^: *p* value for association
PACLR: Poland Anterior Cruciate Ligament Rupture
*P*^b^: *p* value for heterogeneity
POL: Poland
PRISMA: Preferred Reporting Items for Systematic Reviews and Meta-Analyses
R: Recessive
RE: Random-effects
RH: Reduced heterogeneity
rs12722: BstUI
rs13946: DpnII
*S*: Number of significant associations
SA: South Africa
SACLR: South Africa Anterior Cruciate Ligament Rupture
SAT: South Africa Achilles Tendinopathy
SE: Standard error
Sx: Sex
TE: Tennis elbow
TLI: Tendon-ligament injury
TUR: Turkey
UK: United Kingdom
UM: Used matching
*var*: Variant
We: Weight
*wt*: Wild-type
