# Supplementary material for: Association of COL5A1 gene polymorphisms and risk of tendon-ligament injuries among Caucasians: a meta-analysis
Source: Sports Med Open. 2018 Oct 22;4:46. doi: 10.1186/s40798-018-0161-0 (PMC6197339; doi:10.1186/s40798-018-0161-0)
Supplement: Supplementary file 1 — Table S1. Quantitative characteristics of the included studies that examined the association of COL5A1 polymorphisms with TLI studies. (DOCX 57 kb) [file 40798_2018_161_MOESM1_ESM.docx]

**Table S1** Quantitative characteristics of the included studies that examined the association of *COL5A1* polymorphisms with TLI studies

|  |  |  |  |  | Genotype Frequencies | | | | | | |  |  |
| --- | --- | --- | --- | --- | --- | --- | --- | --- | --- | --- | --- | --- | --- |
|  |  | Sample Sizes | | | Case | | |  | Control | | | |  |
|  | First Author Country | Case | Control | Total | ww | wv | vv | maf | ww | wv | vv | maf | HWE |
|  |  |  |  |  |  |  |  |  |  |  |  |  | *p-*value |
| **rs12722** (1,234 cases/1,667 controls) 99^a^ | | | |  | TT | CT | CC |  | TT | CT | CC |  |  |
| 1 | Altinisik TU | 154 | 195 | 349 | 99 | 50 | 5 | 0.20 | 99 | 89 | 7 | 0.26 | **0.02** |
| 2 | Brown UK | 100 | 100 | 200 | 26 | 46 | 28 | 0.71 | 27 | 51 | 22 | 0.48 | 0.82 |
| 3 | Mokone SA | 222 | 258 | 480 | 169 | 40 | 13 | 0.15 | 173 | 77 | 8 | 0.18 | 0.87 |
| 4 | O'Connell SA | 224 | 231 | 455 | 72 | 121 | 31 | 0.41 | 65 | 114 | 52 | 0.47 | 0.88 |
| 5 | O'Connell POL | 91 | 143 | 234 | 32 | 44 | 15 | 0.41 | 38 | 75 | 30 | 0.47 | 0.53 |
| 6 | Posthumus SA | 127 | 216 | 343 | 42 | 68 | 17 | 0.38 | 62 | 110 | 44 | 0.46 | 0.71 |
| 7 | September AU | 85 | 210 | 295 | 17 | 58 | 10 | 0.46 | 75 | 85 | 50 | 0.44 | **0.01** |
| 8 | September SA | 93 | 131 | 224 | 34 | 47 | 12 | 0.38 | 39 | 55 | 37 | 0.49 | 0.07 |
| 9 | Stepien-Slodkowska POL | 138 | 183 | 321 | 48 | 66 | 24 | 0.41 | 53 | 91 | 39 | 0.46 | 0.995 |
| **rs13946** (546 cases/934 controls) 96^a^ | | | |  | TT | CT | CC |  | TT | CT | CC |  |  |
| 1 | Altinisik TU | 154 | 195 | 349 | 49 | 77 | 28 | 0.41 | 94 | 81 | 20 | 0.31 | 0.68 |
| 2 | Posthumus SA | 76 | 214 | 290 | 34 | 40 | 2 | 0.29 | 113 | 90 | 11 | 0.26 | 0.20 |
| 3 | September AU | 85 | 210 | 295 | 53 | 30 | 2 | 0.20 | 115 | 78 | 17 | 0.27 | 0.34 |
| 4 | September SA | 93 | 132 | 225 | 54 | 35 | 4 | 0.23 | 76 | 41 | 15 | 0.26 | **0.02** |
| 5 | Stepien-Slodkowska POL | 138 | 183 | 321 | 69 | 57 | 12 | 0.29 | 84 | 88 | 11 | 0.30 | 0.05 |
| **rs71746744** (191 cases/299 controls) 58^a^ | | | |  | +/+ | +/- | -/- |  | +/+ | +/- | -/- |  |  |
| 1 | Abraham AU | 40 | 97 | 137 | 25 | 12 | 3 | 0.23 | 45 | 37 | 15 | 0.35 | 0.12 |
| 2 | Abraham SA | 51 | 101 | 152 | 31 | 19 | 1 | 0.21 | 43 | 49 | 9 | 0.33 | 0.34 |
| 3 | Brown SA | 100 | 101 | 201 | 50 | 42 | 8 | 0.29 | 41 | 52 | 8 | 0.39 | 0.12 |
| **rs16399** (120 cases/254 controls) 44^a^ | | | |  | -/- | +/- | +/+ |  | -/- | +/- | +/+ |  |  |
| 1 | Abraham AU | 52 | 121 | 173 | 33 | 17 | 2 | 0.20 | 54 | 47 | 20 | 0.36 | 0.09 |
| 2 | Abraham SA | 68 | 133 | 201 | 37 | 28 | 3 | 0.25 | 59 | 62 | 12 | 0.32 | 0.45 |
| **rs3196378** (270 cases/520 controls) 76^a^ | | | |  | AA | AC | CC |  | AA | AC | CC |  |  |
| 1 | Brown SA | 100 | 100 | 200 | 32 | 45 | 23 | 0.46 | 27 | 47 | 26 | 0.50 | 0.55 |
| 2 | September AU | 85 | 210 | 295 | 8 | 64 | 13 | 0.53 | 37 | 118 | 55 | 0.63 | 0.06 |
| 3 | September SA | 85 | 210 | 295 | 17 | 50 | 18 | 0.51 | 53 | 104 | 53 | 0.50 | 0.89 |
|  |  |  |  |  |  |  |  |  |  |  |  |  |  |

TU: Turkey; UK: United Kingdom; SA: South Africa; AU: Australia; POL: Poland; w: wild-type; v: variant; TLI: tendon-ligament injury; maf: minor allele frequency; HWE: Hardy-Weinberg Equilibrium; Values in bold indicate those *p*-values that significantly departed from HWE; Superscript a: statistical power (%) for the combined studies obtained from the aggregate sample sizes for cases and controls in each *COL5A1* polymorphism. Statistical power was calculated with the G*Power program (http://www.psycho.uni-duesseldorf.de/aap/projects/gpower) as probability of detecting an association between the *COL5A1* polymorphisms and TLI assuming small effect size (d = 0.20) and α level of 0.05.
